# Jasmonate inhibits adventitious root initiation through repression of *CKX1* and activation of RAP2.6L transcription factor in Arabidopsis

**DOI:** 10.1093/jxb/erab358

**Published:** 2021-07-30

**Authors:** Asma Dob, Abdellah Lakehal, Ondrej Novak, Catherine Bellini

**Affiliations:** 1 Umeå Plant Science Centre, Department of Plant Physiology, Umeå University, SE-90736 Umeå, Sweden; 2 Laboratory of Growth Regulators, Faculty of Science, Palacký University and Institute of Experimental Botany, Academy of Sciences of the Czech Republic, 78371 Olomouc, Czech Republic; 3 Umeå Plant Science Centre, Department of Forest Genetics and Plant Physiology, Swedish University of Agricultural Sciences, Umeå, Sweden; 4 Institut Jean-Pierre Bourgin, INRAE, AgroParisTech, Université Paris-Saclay, FR-78000 Versailles, France; 5 University of Maryland, USA

**Keywords:** Adventitious roots, Arabidopsis, CKX1, cytokinins, jasmonate, light, MYC2, RAP2.6L, vegetative propagation

## Abstract

Adventitious rooting is a *de novo* organogenesis process that enables plants to propagate clonally and cope with environmental stresses. Adventitious root initiation (ARI) is controlled by interconnected transcriptional and hormonal networks, but there is little knowledge of the genetic and molecular programs orchestrating these networks. Thus, we have applied genome-wide transcriptome profiling to elucidate the transcriptional reprogramming events preceding ARI. These reprogramming events are associated with the down-regulation of cytokinin (CK) signaling and response genes, which could be triggers for ARI. Interestingly, we found that CK free base (iP, *t*Z, *c*Z, and DHZ) content declined during ARI, due to down-regulation of *de novo* CK biosynthesis and up-regulation of CK inactivation pathways. We also found that MYC2-dependent jasmonate (JA) signaling inhibits ARI by down-regulating the expression of the *CYTOKININ OXIDASE/DEHYDROGENASE1* (*CKX1*) gene. We also demonstrated that JA and CK synergistically activate expression of the transcription factor RELATED to APETALA2.6 LIKE (RAP2.6L), and constitutive expression of this transcription factor strongly inhibits ARI. Collectively, our findings reveal that previously unknown genetic interactions between JA and CK play key roles in ARI.

## Teaser

Genome-wide analysis in Arabidopsis revealed that light controls CK homeostasis during early stages of adventitious root initiation (ARI). CK and JA cooperatively inhibit ARI by inducing the transcription of the transcription factor RAP2.6L.

## Introduction

Unlike most animals, plants have evolved a remarkable capacity to regenerate new organs post-embryonically. A prime example of this feature is the *de novo* regeneration of adventitious roots (ARs), which form from differentiated cells of non-root organs (e.g. stems, hypocotyls, or leaves) (Ikeuchi *et al.*, 2019). They can also form during the intrinsic development of many plant species, including both monocotyledons (in which they are often called crown roots) and dicotyledons (Geiss *et al.*, 2018; Lakehal and Bellini, 2019). In addition to participating in uptake of water and nutrients, ARs formed either during plants’ general development or in response to environmental stresses may contribute to anchoring the plant in the soil (Nam *et al.*, 2020). Their formation in response to other environmental stresses may also enhance a plant’s ability to cope with flooding, waterlogging, wounding, or nutrient deficiency (Steffens and Rasmussen, 2016). Moreover, adventitious rooting is a key step in vegetative (or clonal) propagation, which is widely used in forestery and agriculture to multiply elite clones relatively quickly and cheaply. However, several plant species with high economic and/or ecological importance have low tendencies to form adventitious roots following cutting, even in the presence of root-promoting phytohormones, such as auxin (Lakehal and Bellini, 2019). Moreover, the adventitious rooting competence of cuttings is influenced by both extrinsic factors (such as light intensity and spectral quality, humidity, and temperature) and intrinsic factors, such as their genetic and epigenetic status and the mother plant’s physiological status, for example age (Bellini *et al.*, 2014).

Numerous studies with various model species including *Arabidopsis thaliana* have shown that AR initiation (ARI), like most organogenesis processes, is tightly controlled by coordinated transcriptional networks involving complex circuits and feedback loops (Lakehal *et al.*, 2019*b*). The underlying genetic and molecular programs are not well understood, but both synergistic and antagonistic hormonal crosstalk are strongly involved in regulation of the transcriptional networks (Bellini *et al.*, 2014; Lakehal and Bellini, 2019; Lakehal *et al.*, 2020*b*).

Auxin signaling promotes ARI by modulating homeostasis of the negative regulator jasmonic acid (JA) in Arabidopsis (Sorin *et al.*, 2005; Gutierrez *et al.*, 2009, 2012). Auxin, perceived by the F-box proteins TRANSPORT INHIBITOR1/AUXIN-SIGNALLING F-BOX PROTEIN (TIR1 and AFB2), triggers degradation of the repressor proteins AUXIN/INDOLE-3-ACETIC ACID 6 (IAA6), IAA9, and IAA17. This then allows AUXIN RESPONSE FACTOR 6 (ARF6) and ARF8 transcription factors to induce expression of GRETCHEN HAGEN3 (GH3.3), GH3.5, and GH3.6 enzymes (Gutierrez *et al.*, 2012; Lakehal *et al.*, 2019*a*). These three enzymes redundantly promote ARI by conjugating JA to amino acids (Gutierrez *et al.*, 2012). Accordingly, genetic analysis has confirmed that MYC2-mediated JA signaling in the xylem pole pericycle cells represses early ARI events (Lakehal *et al.*, 2020*a*). Moreover, JA represses ARI through transcriptional activation of genes encoding the APETALA2/ETHYLENE RESPONSE FACTOR115 (ERF115) transcription factor and its closely related paralogs ERF113 (also known as RELATED to APETALA2.6 LIKE or RAP2.6L) and ERF114 (Lakehal *et al.*, 2020*a*). These three transcription factors control several developmental programs, including wound healing, shoot and root regeneration, callus formation, stem cell replenishment, and ARI (Che *et al.*, 2006; Heyman *et al.*, 2013, 2016; Ikeuchi *et al.*, 2017; Kong *et al.*, 2018; Zhou *et al.*, 2019; Lakehal *et al.*, 2020*a*). The mechanisms enabling them to play precise roles in multiple processes are still poorly understood, but they may act through preferential activation or repression of downstream targets in a context-dependent manner. For example, *ERF115* inhibits ARI by promoting expression of the *ATP/ADP ISOPENTENYLTRANSFERASE 3* (*IPT3*) gene (Lakehal *et al.*, 2020*a*), which encodes an enzyme that catalyzes the first, rate-limiting step in *de novo* cytokinin (CK) biosynthesis (Miyawaki *et al.*, 2006). CKs are adenine-derived phytohormones that participate in the control of numerous developmental processes, including adventitious rooting (Kieber and Schaller, 2014). Like JA, CKs inhibit ARI in a dose-dependent manner and their action seems to be evolutionarily conserved among various plant species (Ramírez-Carvajal *et al.*, 2009; Mao *et al.*, 2019; Lakehal *et al.*, 2020*a*). Notably, both JA and CK pathways have been shown to be regulated by environmental cues such as light and wounding (and thus cutting). JA rapidly and transiently accumulates at cutting sites and its peak is followed by an accumulation of CK species (Ikeuchi *et al.*, 2017), raising the hypothesis that these two hormones might have interactive effects during ARI. Thus, previous studies have provided substantial information about the roles of JA and CKs, but not the interplay between them during ARI, or even whether they directly interact during the process. To acquire such information, we have examined their interactions. As reported here, we found that dark to light shifts decrease CK free base contents and hence repress CK signaling outputs. We also show that JA inhibits ARI through transcriptional repression of the *CKX1* gene, which encodes the CK-degrading enzyme CK oxidase/dehydrogenase. Moreover, JA and CK additively induce expression of the *RAP2.6L* transcription factor gene, which is a negative regulator of ARI.

## Materials and methods

### Plant material

Seeds of the *ninja-1myc2-322B* double mutant (Gasperini *et al.*, 2015), *coi1-16* (Ellis and Turner, 2002), *tir1-1afb2-3* (Parry *et al.*, 2009), *35S:CKX1* (Werner *et al.*, 2003), and *35S:RAP2.6L* (Krishnaswamy *et al.*, 2011) lines were respectively provided by E.E. Farmer (University of Lausanne, Switzerland), L. Pauwels (VIB/PSB, Ghent, Belgium), M. Estelle (UCSD, San Diego, CA, USA), T. Schmülling (Freie Universität Berlin, Germany), and N. Kav (University of Alberta, Edmonton, Canada). The triple mutant *ninja-1myc2-322B-35S:CKX1* was generated by crossing *ninja-1myc2-322B* and *35S:RAP2.6L* plants. The *A. thaliana* ecotype Col-0 was used as the wild type and background for all the mutants and the *35S:CKX1* transgenic line, except the *35S:RAP2.6L* transgenic line, which was in the Ws-4 background.

### Adventitious and lateral root phenotyping and growth conditions

Seedlings were grown in adventitious root-inducing conditions as previously described (Sorin *et al.*, 2005; Gutierrez *et al.*, 2009, 2012; Lakehal *et al.*, 2019*a*). Briefly, they were grown in dark conditions until their hypocotyls were 6–7 mm long then transferred to long-day conditions. Numbers of ARs (primordia and emerged) were counted under a binocular stereomicroscope 7 d after transferring the seedlings to light conditions. Numbers of emerged lateral roots were counted on the same day from scanned plates. The seedlings’ primary root lengths were measured from scanned plates using ImageJ software (Schindelin *et al.*, 2012), and their lateral root densities (lateral root numbers to primary root length ratios) were calculated.

### Gene expression experiments

#### Sample preparation

To check the effect of JA on *CKX1* expression, total RNA was extracted from whole wild-type (Col-0) and mutant *coi1-16* seedlings, which were grown under long-day conditions for 5 d post-germination then moved to sterile liquid media for overnight acclimation. The next day, the seedlings were either treated with JA (J2500, Sigma) at selected concentrations (2, 10, 20, or 50 μM) or mock treated.

To characterize *CKX1* expression in the *ninja-1myc2-322B* double mutant and Col-0 seedlings during ARI, total RNA was extracted from dissected hypocotyls of etiolated seedlings. The seedlings were grown under AR-inducing conditions as described above; that is, grown in the dark until their hypocotyls were 6–7 mm long (T0) then transferred to light for either 9 h (T9) or 24 h (T24).

To examine effects of CK and JA on *RAP2.6L* expression, total RNA was extracted from etiolated wild-type (Col-0) seedlings. As *RAP2.6L* expression is down-regulated by light, we decided to check its expression in dark-grown seedlings. The seedlings were first etiolated in the dark until their hypocotyls were ~6 mm long then transferred to liquid media for acclimation overnight. The next day they were treated with either *trans*-zeatin (*t*Z; 1 or 10 μM), JA (25 μM), both *t*Z (1 μM or 10 μM) and JA (25 μM), or mock treated.

#### RNA isolation and cDNA synthesis

Total RNA was extracted using an RNAqueous® Total RNA Isolation kit (Ambion™) then treated with DNase I using a DNA*free* Kit (Ambion™) to remove contaminating DNA. cDNA was synthesized by reverse transcription of the RNA using a SuperScript II Reverse transcriptase kit (Invitrogen) with anchored oligo(dT)_18_ primers. All the steps were performed according to the kit manufacturer’s instructions.

#### Quantitative RT-PCR

Transcript amounts were quantified by quantitative real-time PCR (qRT-PCR), using triplicate reaction mixtures containing 5 μl of cDNA, 0.5 μM of both forward and reverse primers, and 1× LightCycler 480 SYBR Green I Master (Roche) (final volume, 20 μl) according to the manufacturer’s instructions. All these experiments were performed with at least two independent biological replicates. Relative amounts of transcripts of the target genes were calculated as previously described (Gutierrez *et al.*, 2009) and regarded as significantly down- or up-regulated if fold differences were ≤0.75 or ≥1.5, respectively, with *P*-values ≤0.05. Reference genes were validated as the most stably expressed genes under our experimental conditions (Gutierrez *et al.*, 2009) using GenNorm software, and the most stable pair were used to normalize the qPCR data. Data obtained using *TIP41* as the reference gene are presented here. Sequences of primers used for all target and reference genes are listed in Supplementary Table S1.

#### CK quantification

For CK quantification, wild-type seedlings were grown under AR-inducing conditions as described above. The seedlings were first etiolated in the dark until their hypocotyls were 6–7 mm long (T0), then transferred to light for either 9 h (T9) or 72 h (T72). Etiolated hypocotyls were collected and rapidly dried on tissue paper then frozen in liquid nitrogen. The samples were stored at –80 °C until further use. Samples were prepared from six biological replicates, each consisting of two technical replicates. CK species (ribotides, ribosides, free bases, and glucosyl conjugates) were quantified as previously described (Lakehal *et al.*, 2020*a*) from 20 mg FW samples using a published methodology (Svačinová *et al.*, 2012; Antoniadi *et al.*, 2015).

### Statistical analysis

GraphPad Prism 8 software (https://www.graphpad.com/) was used to perform all the statistical analysis, and each statistical analysis is described in detail in its corresponding figure legend.

## Results

### Dark–light transition triggers profound transcriptional reprogramming

**Fig. 1.**
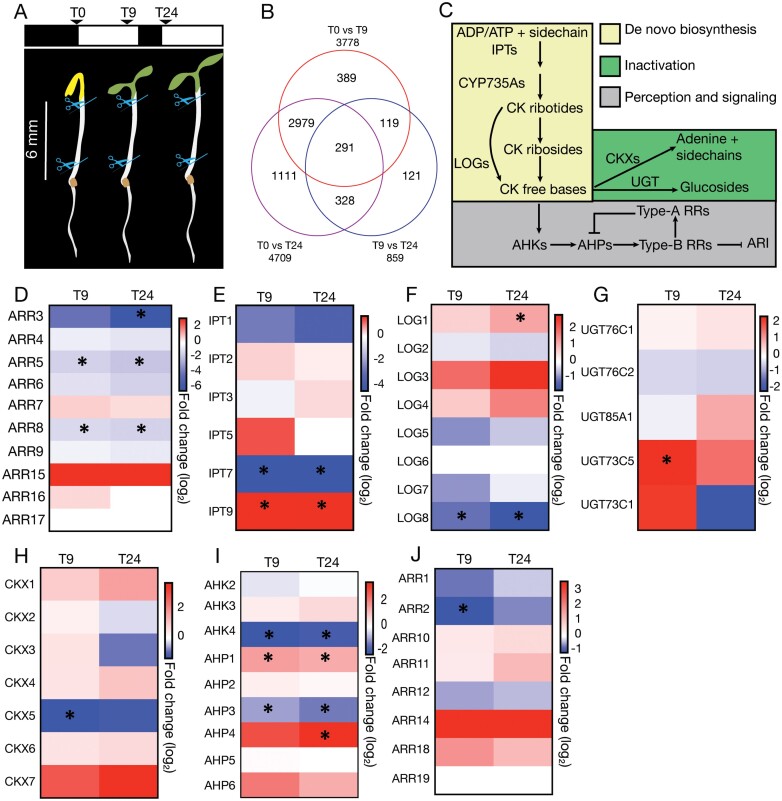
Dark–light transition affects CK pathways. (A) Representative scheme of the experimental set-up used to collect samples for RNA sequencing. Seedlings were grown in the dark until their hypocotyls were 6–7 mm long (T0) then shifted to the light for either 9 h (T9) or 24 h (T24). (B) Venn diagram showing numbers of differentially expressed genes during ARI in wild-type (Col-0) seedlings. (C) Illustrative model of CK pathways. (D–J) Heatmaps of the genes involved in the CK pathways: (D) response, (E and F) *de novo* biosynthesis, (G) inactivation, (H) irreversible cleavage, and (I and J) signaling. Heatmaps represent fold changes (log2) in transcript abundance in wild-type seedlings. Blue and red colors indicate down-regulated and up-regulated expression, respectively, at T9 or T24 (9 h and 24 h after transfer to light, at T0 respectively), relative to T0. Asterisks indicate significant differences.

To uncover the transcriptional reprogramming events associated with ARI in Arabidopsis, we re-analyzed our publicly available transcriptome dataset (Lakehal *et al.*, 2020*a*). This dataset was generated by sequencing RNA extracted from etiolated hypocotyls of wild-type (Col-0) seedlings at T0 (just before moving them into the light), at T9 (9 h after the transfer), and at T24 (24 h after the transfer) (Fig. 1A). This analysis detected 3778 differentially expressed genes (DEGs), 2480 of which were up-regulated and 1298 down-regulated at T9 compared with T0 (Fig. 1B; Supplementary Table S2). It also detected 4709 DEGs between T0 and T24, 3001 of which were up-regulated and 1708 down-regulated at T24 compared with T0. Moreover, 859 were differentially expressed (433 up-regulated and 426 down-regulated) at T24 compared with T9 (Fig. 1B; Supplementary Tables S3, S4). These data clearly show that shifting such seedlings from dark to light causes profound transcriptional reprogramming in the hypocotyls, which probably involves changes in the expression of genes that regulate the cell fate decision programs leading to ARI.

### CK pathways are reprogrammed during the early stages of ARI

We have previously shown that CKs repress ARI in Arabidopsis (Lakehal *et al.*, 2020*a*), but the mechanisms modulating CK pathways during this process have remained unclear. To clarify them, we first checked expression profiles of type-A *ARABIDOPSIS RESPONSE REGULATOR* (*ARR*) genes, which are used as proxies for CK response status (Fig. 1C) (Brenner *et al.*, 2005; Bhargava *et al.*, 2013; Kieber and Schaller, 2018). Interestingly, we found that three type-A *ARR* genes (*ARR3*, *ARR5*, and *ARR8*) were down-regulated upon shifting seedlings from dark to light, suggesting that CK signaling is repressed during the early stages of ARI (Fig. 1C, D). To understand the causes of the repression of CK signaling, we examined expression profiles of genes involved in CK homeostasis and signaling pathways (Kieber and Schaller, 2014, 2018) (Fig. 1C). We found that several key genes were down-regulated upon shifting seedlings from dark to light. These included *IPT7* and *LONELY GUY* (*LOG8*) involved in *de novo* CK synthesis, *ARABIDOPSIS HISTIDINE KINASE 4* (*AHK4*) and *HISTIDINE-CONTAINING PHOSPHOTRANSMITTER3* (*AHP3*) involved in perception and transduction, and type-B *ARR2* involved in transcriptional regulation. In addition, *UDP-GLUCOSYL TRANSFERASE 73C5* (*UGT73C5*), encoding an enzyme catalyzing CK inactivation (Hou *et al.*, 2004), was up-regulated (Fig. 1E–J; Supplementary Tables S2–S4). These results suggest that repression of CK signaling may be due to a reduction in CK biosynthesis and an increase in CK inactivation. However, we also found that *IPT9*, *LOG1*, *AHP1*, and *AHP4* were up-regulated and *CYTOKININ OXIDASE/DEHYDROGENASE5* (*CKX5*) was down-regulated upon shifting the seedlings from dark to light, suggesting the possible existence of compensatory mechanisms (Fig. 1E–I; Supplementary Tables S2–S4).

Taken together, these results suggest that dark to light transition generates cues that repress CK signaling and thereby de-repress the gene expression programs leading to ARI.

### Dark–light transition decreases *de novo* biosynthesis of CKs and increases their inactivation

**Fig. 2.**
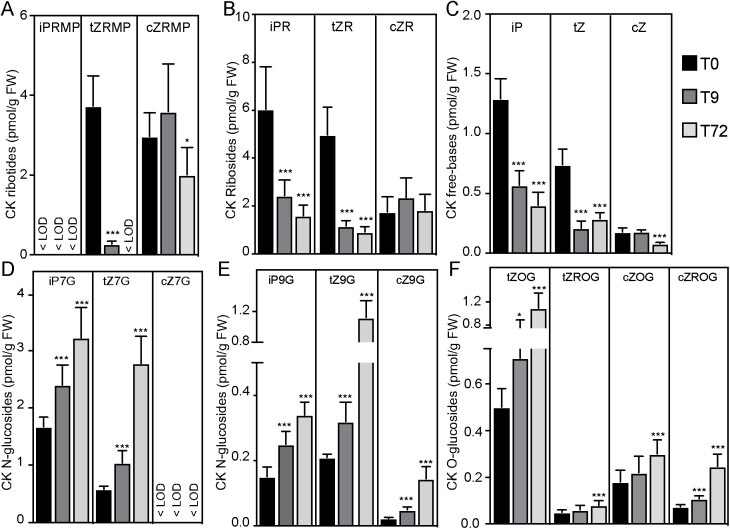
Dark–light transition affects CK homeostasis. Contents in wild-type (Col-0) seedlings grown in the dark until their hypocotyls were 6–7 mm long (T0) then shifted to the light for either 9 h (T9) or 72 h (T72) of: (A) CK ribotides [isopentenyladenine riboside-5′-monophosphate (iPRMP), *trans*-zeatin riboside-5′-monophosphate (*t*ZRMP), *cis*-zeatin riboside-5′-monophosphate (*c*ZRMP)]; (B) CK ribosides [isopentenyladenine riboside (iPR), *trans*-zeatin riboside (*t*ZR), *cis*-zeatin riboside (*c*ZR)]; (C) CK free bases [isopentenyladenine (iP), *trans*-zeatin (*t*Z), *cis*-zeatin (*c*Z)]; (D and E) CK *N*-glucosides [isopentenyladenine-7-glucoside (iP7G), *trans*-zeatin-7-glucoside (*t*Z7G), *cis*-zeatin-7-glucoside (*c*Z7G), isopentenyladenine-9-glucoside (iP9G), *trans*-zeatin-9-glucoside (*t*Z9G), *cis*-zeatin-9-glucoside (*c*Z79G)]; and (F) CK *O*-glucosides [*trans*-zeatin-*O*-glucoside (*t*ZOG), *trans*-zeatin riboside-*O*-glucoside (*t*ZROG), *cis*-zeatin-*O*-glucoside (*c*ZOG), *cis*-zeatin riboside-*O*-glucoside (*c*ZROG)]. *, **, and *** indicate significant differences (0.05>*P*>0.01, 0.01>*P*>0.001, and *P*<0.001, respectively) at T9 and T72 relative to T0 according to ANOVA with *t*-tests. Means (bars) and SDs (whiskers). <LOD indicates under the limit of detection.

As dark–light transition led to a repression of CK responses that was coupled with down-regulation of genes involved in *de novo* biosynthesis and up-regulation of a gene involved in CK inactivation, we hypothesized that down-regulation of CK signaling is caused by reduction in CK free base content. To test this hypothesis, we quantified the CK free bases: isopentenyladenine (iP), *t*Z, *cis*-zeatin (*c*Z), and dihydrozeatin (DHZ). The CK free bases are the active forms that bind to CK receptors and thereby trigger the downstream signaling events (Kieber and Schaller, 2014). We also quantified their riboside precursors (iPR, *t*ZR, *c*ZR, and DHZR), corresponding 5-monophosphate ribotides (iPRMP, *t*ZRMP, *c*ZRMP, and DHZMP), and glucosyl conjugates: CK *N*-glucosides (iP7G, *t*Z7G, *c*Z7G, DHZ7G, iP9G, *t*Z9G, *c*Z9G, and DHZ9G) and CK *O*-glucosides (*t*ZROG*, t*ZOG, *c*ZROG, *c*ZOG, DHZROG, and DHZOG). Glucosyl conjugates (*N*-glucosides and *O*-glucosides) are inactive and the CK *N*-glucosides are even thought to be irreversibly inactive (Kieber and Schaller, 2014), except for tZ7G and tZ9G, which may be reactivated by conversion to *t*Z (Hošek *et al.*, 2020). The CK contents of the seedlings were quantified under the same conditions and at the same time points as RNA in the RNA sequencing experiments (Fig. 1A), except T72 (72 h after transferring etiolated seedlings to light) instead of T24 to cover a wider developmental window of AR development. We found that *t*ZRMP, *t*ZR, *t*Z, iPR, and iP contents all declined after moving the seedlings to light (Fig. 2A–C). We also observed slight decreases in *c*ZRMP and *c*Z at T72 compared with T0 (Fig. 2A–C). Moreover, the dark–light transition increased the conjugation process, as manifested by increases in iP7G, iP9G *t*Z7G, *t*Z9G, *c*Z9G, *t*ZOG, *t*ZROG, *c*ZOG, and *c*ZROG (Fig. 2D–F). Interestingly, we also observed reductions in DHZR and DHZ, accompanied by increases in DHZ7G, DHZ9G, and DHZOG, after moving the seedlings to light (Supplementary Fig. S1A–E). Thus, although their biological significance and exact role are not yet well understood, it seems that DHZ-type CKs might also play a role in ARI.

Collectively, these data indicate that the dark–light transition has a dual effect on CK homeostasis, both repressing the *de novo* CK biosynthesis pathways and enhancing the inactivation pathways. Repressing CK free bases below a certain threshold probably triggers the ARI process and subsequently allows development of AR primordia.

### TIR1/AFB2-dependent auxin signaling partly controls the decrease of CK content upon dark–light transition

Light is known to control auxin homeostasis and signaling (Halliday *et al.*, 2009). Because auxin has been shown to negatively control CK biosynthesis (Nordström *et al.*, 2004; Cheng *et al.*, 2013; Zhang *et al.*, 2018), we wondered whether the down-regulation of CK content during dark–light transitions is mediated by auxin signaling. To test this possibility, we quantified the content of total CK, total CK ribosides, total CK *N*-glucosides, and total CK *O*-glucosides in the wild type and in the auxin receptor double mutant *tir1-1afb2-3*. The double mutant *tir1-1afb2-3* is insensitive to auxin (Parry *et al.*, 2009) and thus produces very few ARs compared with the WT (Lakehal *et al.*, 2019*a*). To obtain a wider picture of the CK status, we first quantified the total CK content in the *tir1-1afb2-3* mutant and in the wild type. Interestingly, at T0, just before shifting the seedlings to the light, the total CK content was slightly lower in the *tir1-1afb2-3* double mutant compared with the wild type. However, after shifting the seedlings to the light, it remained stable in *tir1-1afb2-3* while it decreased in the wild type (Supplementary Fig. S2A). Consistently, we observed no significant variation of the total CK ribosides, which are precursors of CK, in the *tir1-1afb2-3* double mutant 9 h after shifting the seedlings to the light, while it was already significantly reduced in the wild type (Supplementary Fig. S2B). These results suggest that TIR1/AFB2-dependent auxin signaling controls CK biosynthesis upon shifting the seedlings from dark to light. We also observed an increase of the total CK conjugate content in both the double mutant *tir1-1afb2-*3 and in the wild type after shifting the seedlings from dark to light. Nevertheless, the amount of CK conjugates was less in the double mutant *tir1-1afb2-*3 compared with the wild type at all the tested time points, suggesting that auxin might also promote CK conjugation (Supplementary Fig. S2C, D). Together, these data suggest that TIR1/AFB2-dependent auxin signaling is involved in the regulation of CK homeostasis upon light–dark transitions, but further research is needed to unravel the exact underlying mechanism.

### JA down-regulates *CKX1* expression in a COI1-dependent manner

**Fig. 3.**
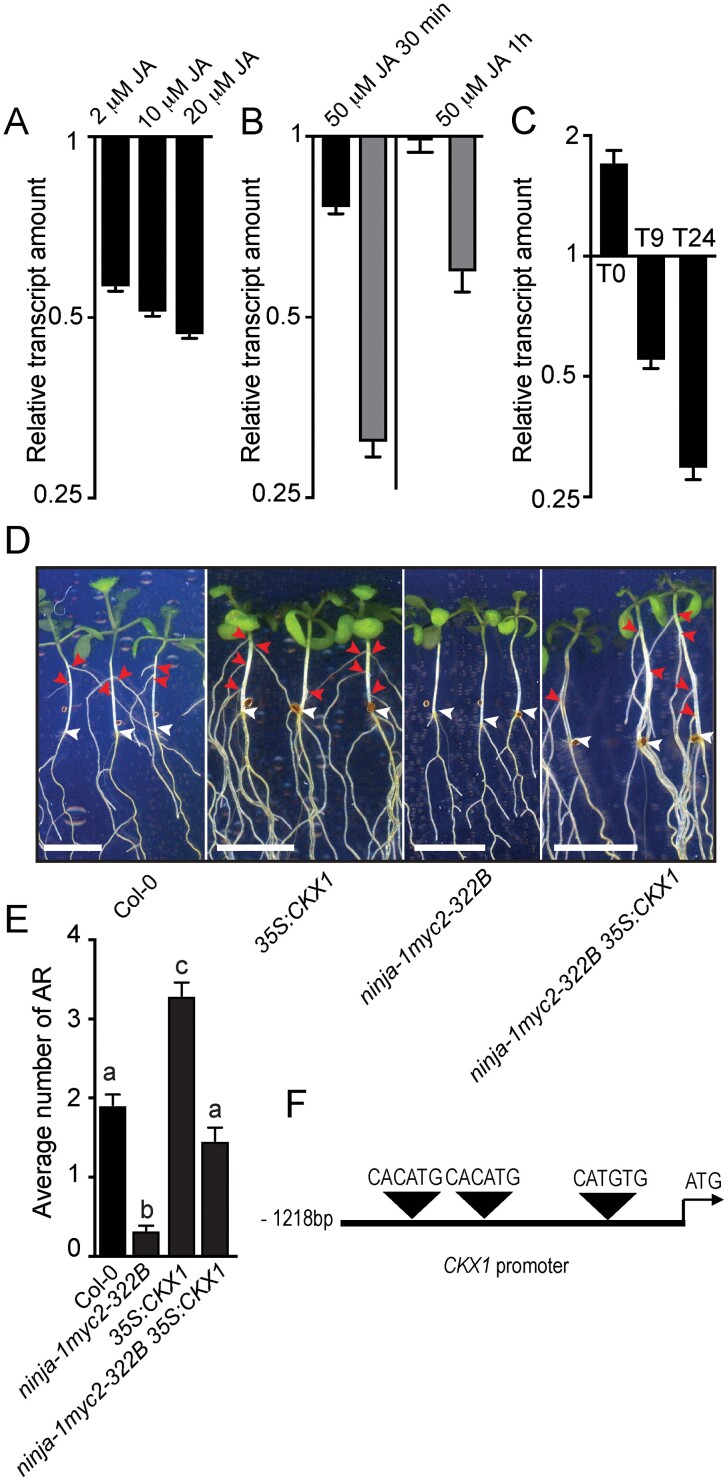
JA inhibits ARI by repressing *CKX1* expression. (A–C) Relative amounts of *CKX1* transcripts measured by qRT-PCR. Means and SEs (indicated by error bars) obtained from three technical replicates. All the qRT-PCR experiments were repeated with at least one other independent biological replicate and gave similar results. (A) Transcripts extracted from 6-day-old wild-type (Col-0) seedlings treated with JA (at the indicated concentrations) for 1 h relative to amounts in mock-treated controls. (B) Transcripts extracted from 6-day-old *coi1-16* mutant (dark bars) or wild-type (gray bars) seedlings treated with 50 μM JA relative to amounts in mock-treated controls. (C) Transcripts extracted from dissected hypocotyls of *ninja-1myc2-322B* double mutant or wild-type seedlings etiolated in the dark until their hypocotyls were 6 mm long (T0) then moved to the light for 9 h (T9) or 24 h (T24) relative to amounts in wild-type (Col-0) seedlings. (D) Representative images showing emerged ARs. Red and white arrowheads indicate emerged ARs and root–hypocotyl junctions, respectively. Scale bars indicate 6 mm. (E) Average numbers of ARs. The non-parametric Kruskal–Wallis test combined with Dunn’s multiple comparison post-test indicates that the *ninja-1myc2-322B35S:CKX1* triple mutant produced significantly more ARs than the *ninja-1myc2-322B* double mutant. Bars and whiskers indicate means and SEs, respectively (*n*>50; *P*<0.0007). (F) Illustrative scheme representing locations of G-box-like motifs in the *CKX1* promoter.

Previous studies have shown that moving seedlings from dark to light reduces JA contents of etiolated hypocotyls (Gutierrez *et al.*, 2012; Lakehal *et al.*, 2020*a*). It was also shown that both JA and CKs repress ARI (Ramírez-Carvajal *et al.*, 2009; Gutierrez *et al.*, 2012; Mao *et al.*, 2019; Lakehal *et al.*, 2020*a*), but not whether they directly interact during this process. In this study, we found that dark to light transition reduces CK contents of etiolated hypocotyls (Fig. 2A–F; Supplementary Fig. S1A–E), raising the possibility that interplay between JA and CK pathways provides a coherent developmental input for ARI. To test the hypothesis that JA might control CK content, we first searched publicly available gene expression datasets (using the Arabidopsis eFP browser) for CK-related genes that are transcriptionally affected by exogenous applications of JA or the JA derivative methyl-jasmonate (MeJA) (Winter *et al.*, 2007). A particularly interesting finding is that *CKX1* expression was weaker in Arabidopsis seedlings treated with MeJA for 1 h or 3 h than in mock-treated counterparts (Supplementary Fig. S3). *CKX1* encodes an endoplasmic reticulum-localized enzyme that catalyzes irreversible degradation of CKs by cleavage of their side chains (Werner *et al.*, 2003; Niemann *et al.*, 2018). The CK cleavage activity of CKX1 was confirmed by using its recombinant protein expressed in *Pichia pastoris* (Kowalska *et al.*, 2010). Interestingly, the *CKX1* gene was slightly up-regulated in hypocotyls of seedlings shifted from dark to light, in accordance with their reduction in content of CK free bases (Fig. 1H). This expression profile was further confirmed by qRT-PCR (Supplementary Fig. S4). These data prompted us to hypothesize that MYC2-dependent JA signaling might control the irreversible cleavage of CKs and thus repress ARI. To test this hypothesis, we first quantified relative amounts of *CKX1* transcripts in wild-type seedlings treated with 2, 10, or 20 μM JA for 1 h. Our data revealed that exogenous applications of JA down-regulated expression levels of *CKX1* in a dose-dependent manner (Fig. 3A), and this down-regulation requires the presence of a functional JA receptor, CORONATINE INSENSITIVE1 (COI1) (Fig. 3B), which triggers transcriptional changes regulated by MYC2.

To obtain more evidence of MYC2’s involvement, we compared amounts of *CKX1* transcripts in *ninja-1myc2-322B* double mutant and wild-type seedlings. *ninja-1* is a loss-of-function mutation (Acosta *et al.*, 2013), whereas *myc2-322B* is a gain-of-function mutation (Gasperini *et al.*, 2015). Combination of these two mutations results in constitutive and enhanced JA signaling (Gasperini *et al.*, 2015; Lakehal *et al.*, 2020*a*). We found that *CKX1* expression was weaker in *ninja-1myc2-322B* double mutants than in wild-type seedlings at T9 and T24, but not at T0 (Fig. 3C). As JA signaling is constitutively enhanced in *ninja-1myc2-322B* double mutants, we expected to observe *CKX1* down-regulation at all sampling time points, but this was not the case at T0. These data suggest that the transcriptional regulation of *CKX1* by MYC2-dependent JA signaling involves other light-dependent factor(s), which require further elucidation.

### *CKX1* action downstream of *MYC2-*dependent JA signaling promotes ARI

To test whether *CKX1* activity downstream of MYC2-dependent JA signaling promotes ARI genetically, we generated triple *35S:CKX1ninja-1myc2-332B* mutants. Double *ninja-1myc2-322B* mutants produced fewer, and overexpressing *35S:CKX1* mutants more, ARs than wild-type seedlings (Fig. 3D, E). These results are consistent with our previous findings (Lakehal *et al.*, 2020*a*). Interestingly, the triple *35S:CKX1 ninja-1myc2-332B* mutants produced similar numbers of ARs to the wild-type seedlings (Fig. 3D, E), indicating that enhancing the irreversible cleavage of CKs in the *ninja1myc2-332B* double mutants is sufficient to suppress the negative effect of JA signaling on ARI. The MYC2 transcription factor preferably binds to G-box or G-box-like *cis*-regulatory elements in the induction or repression of its downstream targets (Godoy *et al.*, 2011). Therefore, we searched for such *cis*-regulatory motifs in the 1.5 kb sequence upstream of the CKX1 gene’s *ATG* translation start codon. Interestingly, we found three G-box-like tetrameric motifs (CACATG, CACATG, and CATGTG), but whether MYC2 represses *CKX1* expression indirectly or directly by binding to its promoter during ARI remains to be investigated (Fig. 3F).

### ARI repression by JA and CKs involves synergistic induction of *RAP2.6L*

To obtain insights into the mechanism whereby JA–CK interplay represses ARI, we searched our transcriptomic database for potential transcription factors that are differentially expressed during the early stages of the process (Fig. 1A). This analysis detected 180 differentially expressed transcription factors, 91 of which were up-regulated and 79 down-regulated at T9 compared with T0 (Supplementary Table S5). It also detected 230 differentially expressed transcription factors between T0 and T24, 143 of which were up-regulated and 87 down-regulated at T24 compared with T0. Moreover, 63 were differentially expressed (39 up-regulated and 24 down-regulated) at T24 compared with T9 (Supplementary Tables S6, S7).

**Fig. 4.**
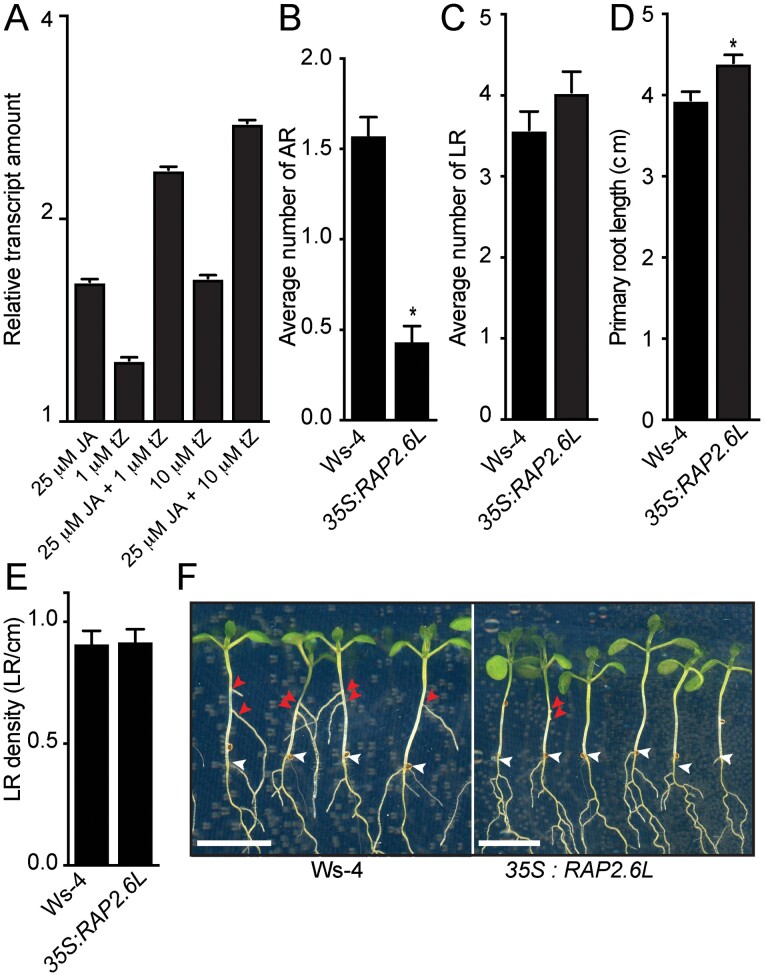
CK and JA synergistically repress ARI through *RAP2.6L* induction. (A) Amounts of *RAP2.6L* transcripts in wild-type seedlings treated with the indicated concentrations of JA and/or *t*Z relative to amounts in mock-treated controls. Means and SEs obtained from three technical replicates. The qRT-PCR experiment was repeated with another independent biological replicate and gave similar results. (B) Average numbers of ARs. The *t*-test indicates that *35S:RAP2.6L* produced significantly fewer ARs than wild-type seedlings (*n*>60; *P*<0.0001). (C) Average numbers of lateral roots (LRs). (D and E) Results of measurements respectively showing that *35S:RAP2.6L* mutants produced longer primary roots than wild-type seedlings (*t*-test: *n*>24, *P*<0.0022), but their lateral root density did not differ (B–E) Bars and whiskers indicate means and SEs, respectively. (F) Representative images showing numbers of emerged ARs. Red and white arrowheads indicate emerged ARs and junction roots, respectively. Scale bars indicate 6 mm.

Interestingly, we retrieved several transcription factor genes with a potential role in *de novo* organogenesis and stem cell regulations, one of which (*RAP2.6L*) was down-regulated at T9 and T24 (Supplementary Tables S5–S7). How *RAP2.6L* is transcriptionally regulated during ARI has remained unclear. We therefore tested whether its expression is affected by exogenous applications of JA and/or CKs. We quantified the relative abundance of this gene’s transcripts in wild-type seedlings treated with 25 μM JA, 1 μM or 10 μM *t*Z, 25 μM JA+1 μM *t*Z, or 25 μM JA+10 μM *t*Z. We found that exogenous applications of both 25 μM JA and 10 μM *t*Z slightly induced expression of *RAP2.6L* (Fig. 4A). Moreover, a combination of JA 25 μM with either 1 μM or 10 μM *t*Z had an additive effect on *RAP2.6L* levels (Fig. 4A). These findings suggest that JA and CK synergistically induce expression of *RAP2.6L*. To further investigate whether enhancing *RAP2.6L* expression is sufficient to repress ARI, we analyzed the AR phenotype in transgenic lines overexpressing *RAP2.6L* (*35S:RAP2.6L*). We found that *35S:RAP2.6L* plants produced dramatically fewer ARs than wild-type (Ws-4) counterparts (Fig. 4B, F). *RAP2.6L* seems to specifically control ARI because lateral root number and density were not affected, although primary roots of *35S:RAP2.6L* seedlings were slightly longer than those of their wild-type counterparts (Fig. 4C–E).

We concluded that CKs and JA may repress ARI by synergistically inducing expression of *RAP2.6L*.

## Discussion

Adventitious rooting is a plastic developmental process that is controlled by multilayered transcriptional cascades involving complex interactions between environmental signals, genetic factors, and cross-linked hormonal networks (Lakehal and Bellini, 2019). It is an intrinsic element of the development of many plant species, and an essential step in mass clonal propagation through stem cuttings in agriculture and forestry (Bellini *et al.*, 2014; Geiss *et al.*, 2018). Environmental factors associated with the capacity to develop ARs include light and wounding (Bellini *et al.*, 2014), and the key phytohormones include CKs, which participate in the control of a plethora of developmental processes (Wybouw and De Rybel, 2019). They also mediate plant responses to diverse environmental cues (Kieber and Schaller, 2014). Thus, there is a clear need for tight spatiotemporal control of dynamic CK pools, especially the free bases, which are mainly governed by the combined action of three interconnected processes: biosynthesis, conjugation, and cleavage (Sakakibara, 2006; Kieber and Schaller, 2014, 2018). Moreover, complex crosstalk with other hormones adds further regulatory layers that play crucial roles in CKs’ precise regulation of multiple physiological and developmental processes (O’Brien and Benková, 2013). Previous studies have firmly established that CKs play a key role in ARI, but provided little knowledge of the mechanisms modulating their pools during early stages of the process.

In the study reported here, we found that dark–light shifts cause profound transcriptional reprogramming of CK pathways in etiolated seedlings (Fig. 1D–J), resulting in substantial reductions in CK contents (Fig. 2A–F) and signaling that are likely triggers of ARI events. Accordingly, reducing CK contents in Arabidopsis either through knocking out key enzymes involved in their *de novo* biosynthesis (as in the *ipt3ipt5ipt7* triple mutant) or by enhancing their irreversible cleavage (as in the *35S:CKX1*-overexpressing line) reportedly leads to significantly more ARs than in wild-type seedlings (Werner *et al.*, 2003; Miyawaki *et al.*, 2006; Lakehal *et al.*, 2020*a*). Blocking the CK perception or signaling pathways (as in *ahk3ahk4*, *arr1arr11*, and *arr1arr11arr12* double and triple mutants) leads to similar increases in AR frequency (Rasmussen *et al.*, 2012; Lakehal *et al.*, 2020*a*). The negative role of CKs in adventitious rooting has also been confirmed in many plant species that rely on vegetative propagation (Lakehal and Bellini, 2019). For example, exogenous application of CK can inhibit AR formation in *Malus domestica* (apple) and *Picea abies* (Norway spruce) cuttings (Mao *et al.*, 2019; Alallaq *et al.*, 2020). Similarly, Ramírez-Carvajal *et al.* (2009) found that overexpressing an active variant of type-B cytokinin *RESPONSE REGULATOR 13* (*PtRR13*) delayed AR formation in *Populus tremula×Populus alba* stem cuttings. The role of CK seems to be evolutionarily conserved because genetic alteration of expression of the *CKX* genes affects AR development in *Oryza sativa* (rice) and *Hordeum vulgare* (barley) (Zhao *et al.*, 2009, 2015; Gao *et al.*, 2014; Jiang *et al.*, 2017; Ramireddy *et al.*, 2018). Moreover, the *CROWN ROOTLESS5* gene, which encodes an AP2/ERF transcription factor, promotes ARI in rice by repressing the CK signaling pathway (Kitomi *et al.*, 2011).

Interactions between light and phytohormones have already been proposed, but no clear mechanistic link between light and CK signaling has been established, and further research is needed to identify the light-related genes mediating the interactions (if any) involved. However, light modulates the homeostasis and signaling of several hormones including auxin (Halliday *et al.*, 2009), which has antagonistic regulatory effects to CKs in diverse developmental programs including ARI (Schaller *et al.*, 2015; Lakehal and Bellini, 2019). It has been shown that auxin may repress CK biosynthesis (Nordström *et al.*, 2004), and the ARF3 transcription factor represses both CK biosynthesis and signaling by down-regulating expression of *IPT*, *LOG*, and *AHK4* genes during flower development (Cheng *et al.*, 2013; Zhang *et al.*, 2018). Hormone analyses reported here suggest that TIR1/AFB2-dependent auxin signaling represses CK biosynthesis during dark–light transitions (Supplementary Fig. S2A–D). However, how TIR1/AFB2-dependent auxin signaling controls this process is still to be determined. It would also be interesting to identify the ARF transcription factor(s) mediating CK biosynthesis during ARI. We have previously shown that the TIR1/AFB2–IAA6/IAA9/IAA17 signaling module controls the transcriptional activity of *ARF6* and *ARF8* genes during ARI (Lakehal *et al.*, 2019*a*). As *ARF6* and *ARF8* expression increases after shifting seedlings from dark to light (Tian *et al.*, 2004; Gutierrez *et al.*, 2012), we are tempted to speculate that ARF6- and ARF8-dependent auxin signaling could impair CK biosynthesis and/or conjugation during dark–light transitions.

Interestingly, we also found that down-regulation of CK content coincided with a reduction in JA content (Gutierrez *et al.*, 2012; Lakehal *et al.*, 2020*a*), suggesting a link between the two hormone pathways. Accordingly, the gene expression data presented here suggest that MYC2-mediated JA signaling represses ARI through transcriptional repression of CKX1 (Fig. 3A–C), which catalyzes irreversible cleavage of CKs (Werner *et al.*, 2003). The transcriptional repression of CKX1 would inevitably lead to the accumulation of CKs, in the absence of reductions in their synthesis. This is consistent with a previous report that exogenous applications of MeJA led to rapid transient accumulation of CKs in *Triticum aestivum* L. (wheat) seedlings (Avalbaev *et al.*, 2016), which was attributed to MeJA both down-regulating *CKX1* expression and impairing the enzymatic activity of its product (Avalbaev *et al.*, 2016).

There have been few demonstrations of the interplay between JA and CKs. However, it has been recently shown that CK signaling promotes JA accumulation through transcriptional activation of key genes in the JA biosynthesis pathway and thus inhibits leaf growth in *Zea mays* (Uyehara *et al.*, 2019, Preprint). In the context of ARI, the possibility that CK might promote JA biosynthesis and/or signaling via a positive feedback loop cannot be excluded. Interestingly, our data revealed that JA and CK additively promote expression of the *RAP2.6L* transcription factor, which is a negative regulator of ARI (Fig. 4A–F). These findings indicate that the crosstalk between JA and CK is complex and might involve parallel pathways that require further elucidation. The *RAP2.6L* gene is a member of the *APETALA2/ETHYLENE RESPONSE FACTOR* subfamily X (Nakano *et al.*, 2006; Heyman *et al.*, 2018) and has been implicated in various developmental and regenerative processes, including shoot meristem proliferation and regeneration, as well as tissue reunion (Che *et al.*, 2006; Yang *et al.*, 2018). Che *et al.* (2006) found that the *RAP2.6L* gene is strongly up-regulated in root explants incubated in CK-rich shoot-inducing medium, and shoot regeneration is severely impaired in *rap2-6l* mutants under these conditions, indicating that *RAP2.6L* is required for CK-mediated shoot regeneration. *RAP2.6L* is also reportedly up-regulated at sites of wounds on inflorescence stems, together with *LIPOXYGENASE2*, which encodes an enzyme involved in JA biosynthesis, suggesting that expression of *RAP2.6L* may be triggered by wound-induced JA (Asahina *et al.*, 2011). These authors also found that exogenous applications of MeJA up-regulated *RAP2.6L* expression (Asahina *et al.*, 2011), in accordance with our findings.

In conclusion, our genome-wide transcriptome analysis showed that dark–light transitions substantially affect CK biosynthesis, homeostasis, and signaling pathways. This modulation is associated with the early events of ARI, highlighting a previously unknown role for CKs in mediating light signaling cues that control ARI. It also identified several new transcriptional regulators with potential roles in AR formation, including the CK/JA-induced transcription factor RAP2.6L. Moreover, our genetic analysis revealed that MYC2-dependent JA signaling participates in CK homeostasis by repressing expression of CKX1 enzyme. Further elucidation of the molecular integration of light and JA/CK signaling cues should provide deeper insights into the complex interactive controls of the timing of the gene expression programs involved in AR development and plasticity.

## Supplementary data

The following supplementary data are available at *JXB* online.

Fig. S1. Dark–light transition causes changes in DHZ-type CKs in etiolated hypocotyls.

Fig. S2. TIR1/AFB2-dependent auxin signaling partly controls CK homeostasis during dark–light transitions.

Fig. S3. MeJA negatively regulates *CKX1* expression.

Fig. S4. *CKX1* expression pattern during ARI.

Table S1. Primers used for qRT-PCR in this study.

Table S2. List of differentially expressed genes (DEGs) in the wild type (Col-0) during ARI (T0 versus T9).

Table S3. List of differentially expressed genes (DEGs) in the wild type (Col-0) during ARI (T0 versus T24).

Table S4. List of differentially expressed genes (DEGs) in the wild type (Col-0) during ARI (T9 versus T24).

Table S5. List of differentially expressed transcription factors (TFs) in the wild type (Col-0) during ARI (T0 versus T9).

Table S6. List of differentially expressed transcription factors (TFs) in the wild type (Col-0) during ARI (T0 versus T24).

Table S7. List of differentially expressed transcription factors (TFs) in the wild type (Col-0) during ARI (T9 versus T24).

erab358_suppl_Supplementary_Figures_S1-S4_Table_S7

erab358_suppl_Supplementary_Tables_S1-S3

erab358_suppl_Supplementary_Tables_S4-S6

## Data Availability

All data supporting the findings of this study are available within the paper and within its supplementary data published online, and are available from the corresponding authors (Abdellah Lakehal and Catherine Bellini), upon request.

## Abbreviations

ARI: adventitious root initiation
CK: cytokinin
DEG: differentially expressed gene
JA: jasmonate
