# Supplementary material for: Jasmonate inhibits adventitious root initiation through repression of CKX1 and activation of RAP2.6L transcription factor in Arabidopsis
Source: J Exp Bot. 2021 Jul 30;72(20):7107–18. doi: 10.1093/jxb/erab358 (PMC8547155; doi:10.1093/jxb/erab358)
Supplement: erab358_suppl_Supplementary_Figures_S1-S4_Table_S7 [file erab358_suppl_supplementary_figures_s1-s4_table_s7.pdf]

Supporting Information for:

Jasmonate inhibits adventitious root initiation through repression of *CKX1* and activation of *RAP2.6L* transcription factor in Arabidopsis

Asma Dob[a,1], Abdellah Lakehal[a,1,2,b], Ondrej Novak[2,3], Catherine Bellini[1,4,b]

[1] Department of Plant Physiology, Umeå Plant Science Centre, Umeå University, SE-90736 Umeå, Sweden

[2] Department of Forest Genetics and Physiology, Umeå Plant Science Centre, Swedish Agriculture University, SE-90183 Umea, Sweden

[3] Laboratory of Growth Regulators, Faculty of Science, Palacký University and Institute of Experimental Botany, Academy of Sciences of the Czech Republic, 78371 Olomouc, Czech Republic

[4] Institut Jean-Pierre Bourgin, INRA, AgroParisTech, CNRS, Université Paris-Saclay, FR-78000 Versailles, France

a These two authors equally contributed to the work.

[b] Corresponding authors:

Prof. Catherine Bellini (Catherine.Bellini@umu.se)

Umeå Plant Science Centre, Department of Plant Physiology,

Umeå University, SE-90736 Umeå, Sweden

Phone: +46907869624

Dr. Abdellah Lakehal (abdellah.lakehal@slu.se)

Umeå Plant Science Centre, Department of Forest Genetics and Physiology,

Swedish Agriculture University, SE-90183 Umea, Sweden

Fig. S1: Dark-light transition causes changes in DHZ in etiolated hypocotyls.

Fig. S2: TIR1/AFB2-dependent auxin signaling partly control CK homeostasis during dark-light transitions.

Fig. S3: MeJA negatively regulates *CKX1* expression.

Fig. S4: *CKX1* expression pattern during ARI.

Supplementary data sets available as excel files

Table S1-S4

Table S1: list of differentially expressed genes (DEGs) in the wild type (Col-0) during ARI (T0 versus T9)

Table S2: list of differentially expressed genes (DEGs) in the wild type (Col-0) during ARI (T0 versus T24)

Table S3: list of differentially expressed genes (DEGs) in the wild type (Col-0) during ARI (T9 versus T24)

Table S4: list of differentially expressed transcription factors (TFs) in the wild type (Col-0) during ARI (T0 versus T9)

Table S5-S6

Table S5: list of differentially expressed transcription factors (TFs) in the wild type (Col-0) during ARI (T0 versus T24)

Table S6: list of differentially expressed transcription factors (TFs) in the wild type (Col-0) during ARI (T9 versus T24)

Table S7: Primers used for qRT-PCR in this study

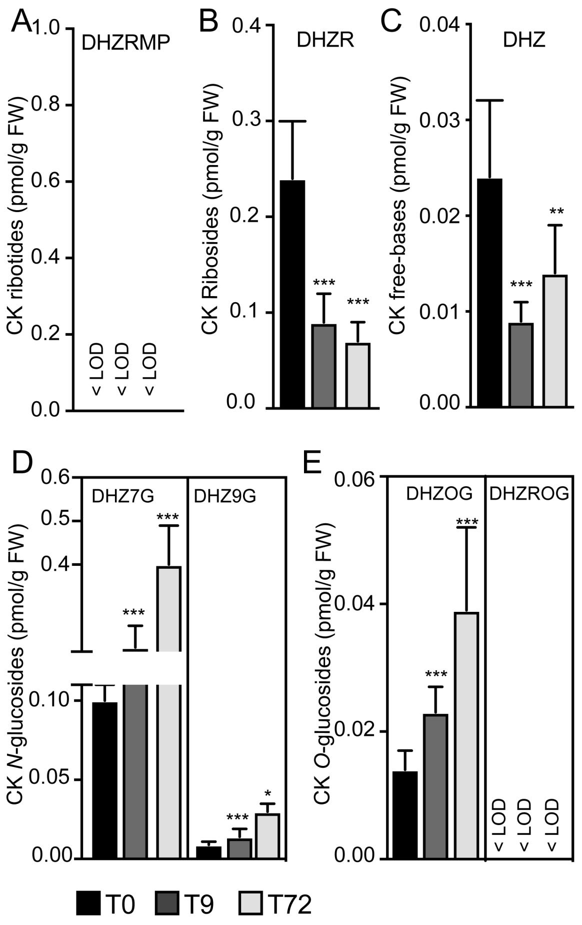

Supplementary Fig. S1: Dark-light transition causes changes in DHZ in etiolated hypocotyls.

CK nucleotides **(A),** CK ribosides **(B)** CK bases **(C),** CK N-glucosides **(D)** and CK O-glucosides **(E)** quantified in hypocotyls dissected from Col-0 seedlings grown in the dark until their hypocotyls were 6 mm long then shifted to the light for 9 h (T9) and 72 h (T72). Means and standard deviations**.** Asterisks indicate significant differences at T9 and T72 versus T0 according to Analysis of Variance with t-tests: *, **, and *** $0.05 > p > 0.01$, $0.01 > p > 0.001$, and $p < 0.001$, respectively). <LOD means under the limit of detection.

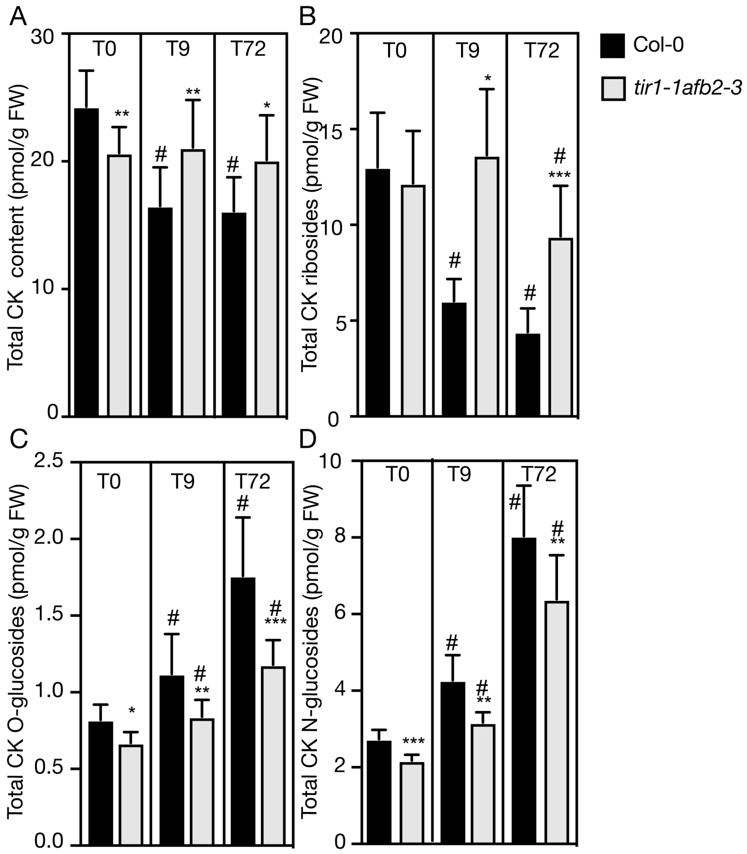

Supplementary Fig. S2: TIR1/AFB2-dependent auxin signaling partly control CK homeostasis during dark-light transitions.

(A) total CK **(B)** total CK ribosides **(C)** CK O-glucosides and **(D)** CK N-glucosides quantified in hypocotyls dissected from Col-0 or *tir1-1afb2-3* seedlings grown in the dark until their hypocotyls were 6 mm long then shifted to the light for 9 h (T9) and 72 h (T72). Means and standard deviations**.** Asterisks indicate significant differences in Col-0 versus *tir1-1afb2-3* at T0, T9 or T72 according to the Analysis of Variance with t-tests:*, **, and *** $0.05 > p > 0.01$, $0.01 > p > 0.001$, and $p < 0.001$, respectively). Dashes # indicate significant differences at T9 and T72 versus T0 in Col-0 or *tir1-1afb2-3* according to the Analysis of Variance.

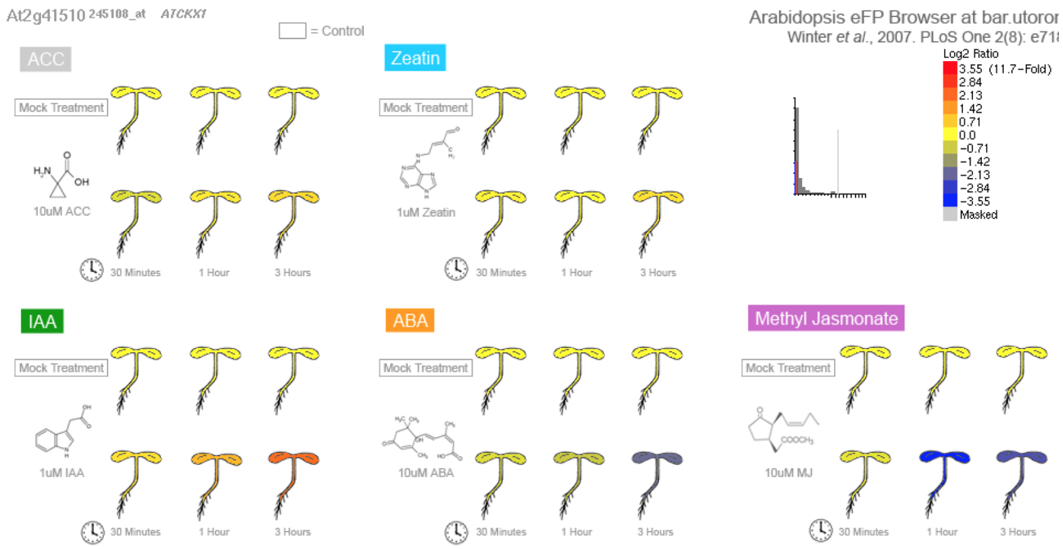

Supplementary Fig. S3: MeJA negatively regulates *CKX1* expression.

Data were retrieved from http://bar.utoronto.ca.

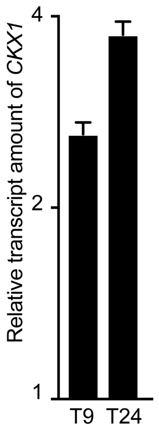

Supplementary Fig. S4: *CKX1* expression pattern during ARI.

Relative amounts of *CKX1* transcripts quantified by qRT-PCR. Amounts of transcripts extracted from dissected hypocotyls of wild-type seedlings etiolated in the dark until the hypocotyl was 6 mm long (T0) then moved to the light for 9 h (T9) or 24 h (T24) relative to amounts detected at T0. Means (bars) and standard errors of the mean (whiskers) obtained from three technical replicates. The experiment was repeated with another independent biological replicate and gave similar results.

Supplementary Table S7: Primers used for qRT-PCR in this study

Primer name	Gene number	Primer forward	Primer reverse
qRT-RAP2.6L	AT5G13330	CAAGGCCCTACTACCACCACAA	GGTCGAGGAGGAGGTGAGTTC
qRT-CKX1	AT2G41510	ATGGATCAGGAAACTGGCAA	AGATGAAAACAAAGTGGATGGAA
qRT-TIP41	At4g34270	GCTCATCGGTACGCTCTTTT	TCCATCAGTCAGAGGCTTCC
